# Improper tube fixation causing a leaky cuff

**DOI:** 10.4103/0974-2700.62125

**Published:** 2010

**Authors:** Babita Gupta, Kamran Farooque, Divya Jain, Rakesh Kapoor

**Affiliations:** Department of Anesthesia and Intensive Care, Jai Prakash Narayan Apex Trauma Centre, All India Institute of Medical Sciences, Ansari Nagar, New Delhi-110 029, India

**Keywords:** Improper fixation, leaky cuff, inflation system

## Abstract

Leaking endotracheal tube cuffs are common problems in intensive care units. We report a case wherein the inflation tube was damaged by the adhesive plaster used for tube fixation and resulted in leaking endotracheal tube cuff. We also give some suggestions regarding the tube fixation and some remedial measures for damaged inflation system.

## INTRODUCTION

A well-secured endotracheal tube (ETT) is essential for safe anesthesia. If ETT is not well secured, there is a danger of either unplanned extubation or advancement of the tube deeper into the trachea. However, improper tube fixation can damage the inflation system and can be a rare cause of leaky ETT cuff, making maintenance of adequate ventilation difficult. We encountered a case wherein the inflation tube was damaged by the adhesive plaster used for tube fixation.

## CASE REPORT

A 33-year-old male patient with the history of motor vehicular accident was brought in the emergency department. On primary survey, his airway was compromised and oxygen saturation with O_2_ by face mask was 78%. His Glasgow goma score was 5; hence to protect the airway, the patient was intubated with 8.0 mm ETT by a resident doctor. The tube was secured with Durapore adhesive plaster at 21 cm at the level of incisors. The adhesive plaster was encircled thrice around the tube; once including the tube and twice excluding the inflation tube. The patient was further investigated and CT scan head revealed a large extradural hematoma with midline shift. The patient was posted for surgical evacuation of extradural hematoma. In operation theatre, it was observed that the ETT cuff was leaking. After checking the exposed part of inflation system, which was intact, it was decided to change the tube. On removing the adhesive plaster, we observed that there was a cut in the inflation tube at the level of entry in ETT. The trachea was reintubated with 8.0 mm ETT. The difficult airway kit, that is, multiple blades, small sized tubes, McCoy laryngoscope, laryngeal mask airway, and tracheostomy kit were kept ready in case of any difficulty in reintubation. On close examination of ETT, we concluded that overextension of the inflation tube lead to the cut in the tubing. Three turns of adhesive plaster once including and twice excluding the inflation tube might be responsible for avulsion of the inflation tube from the tracheal tube. Rest of surgery was uneventful. The patient was on ventilator for 4 days. He was discharged on day 12 after surgery with mild cognitive dysfunction.

## DISCUSSION

A leaking endotracheal cuff may make maintenance of adequate ventilation difficult, fail to protect against aspiration, and make surgery difficult. A defect in the cuff or the inflation system, i.e., inflation tube, pilot balloon, or the valve may be the reasons of leaking cuff. When one is faced with this problem, the best solution is to have the patient reintubated as soon as possible. This is especially true if the patient is mechanically ventilated, because little tidal volume will be delivered while the cuff is unpressurised and deflated. However, extubation, manual ventilation, and reintubation should be avoided, if possible, in certain situations[1]:

- When a mechanically ventilated patient is receiving a high oxygen percentage and/or PEEP level
- Anticipated difficult intubation—it is very important to assess the airway and identify difficult intubation to avoid any catastrophe. The LEMON scale is one of the available predictive methods to assess difficult intubation.[2] The score with a maximum of 10 points is calculated by assigning 1 point for each of the following LEMON criteria
- L = Look externally for any characteristics that are known to cause difficult intubation (facial trauma, large incisors, beard or moustache, large tongue)
- E = Evaluate the 3-3-2 rule (incisor distance, 3 finger breadths; hyoid-mental distance, 3 finger breadths; thyroid to floor of mouth distance, 2 finger breadths)
- M = Mallampati classification; used to visualize the hypopharynx.[3,4] It is not very useful in emergency situations
- O = Obstruction which makes laryngoscopy and intubation difficult (presence of any condition like epiglottitis, peritonsillar abscess, trauma)
- N = Neck mobility (limited neck mobility)

Patients in the difficult intubation group have higher LEMON scores.[2,5]

- When a patient has had upper airway trauma or recent surgery
- When vomiting and aspiration are likely
- When patient has had a recent tracheostomy
- When trained personnel or intubation equipment are not available

**Figure 1:**
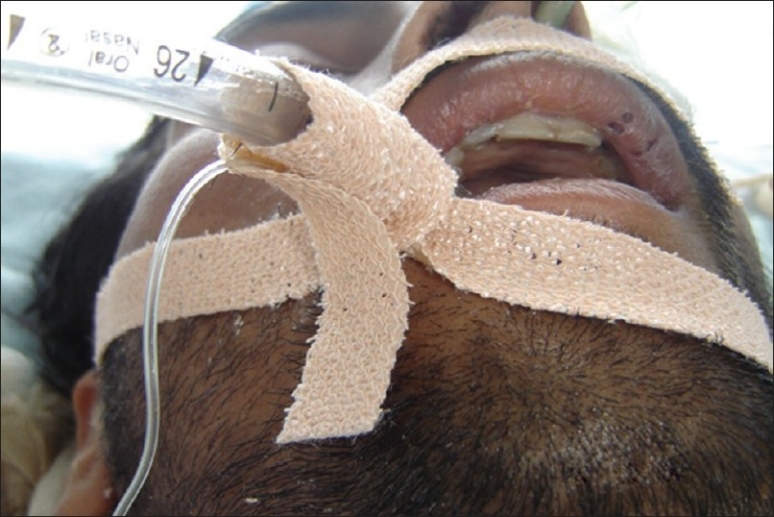
Incorrect endotracheal tube fixation showing multiple overlaps

**Figure 2:**
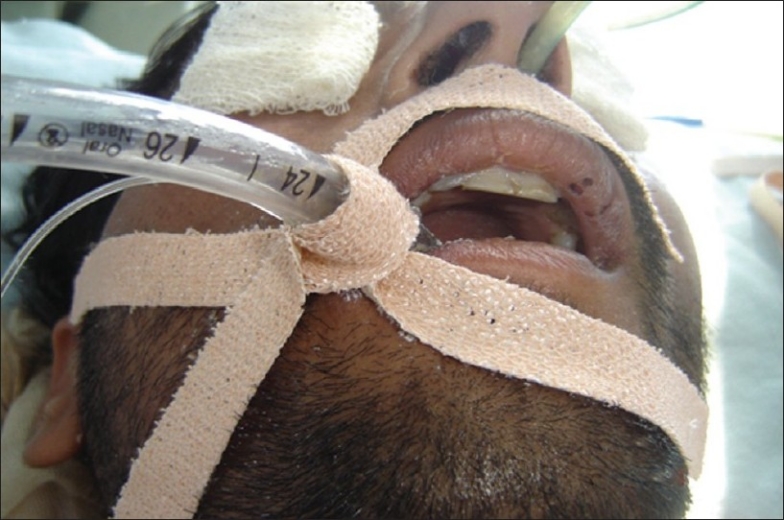
Basic principle of traction and countertraction while securing the endotracheal tube

**Figure 3:**
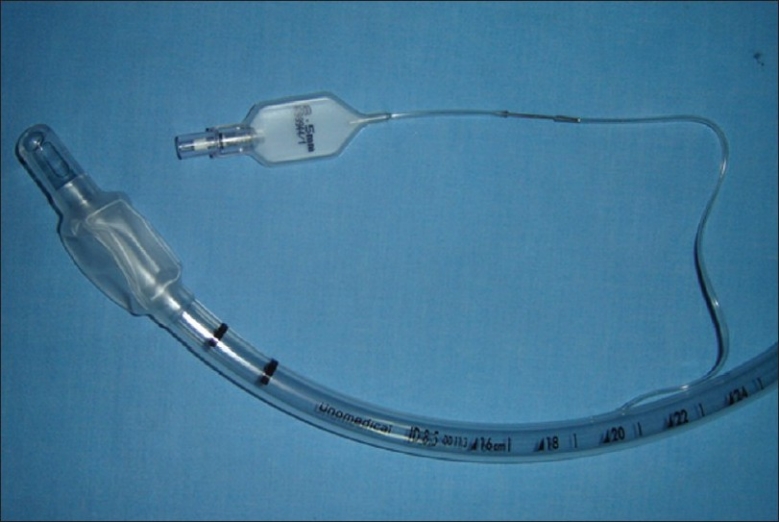
Inflation tube of an unused endotracheal tube joined to remnant section of the inflation tube on the patient's endotracheal tube using a 0.5-1 inch section of a standard hypodermic needle

**Figure 4:**
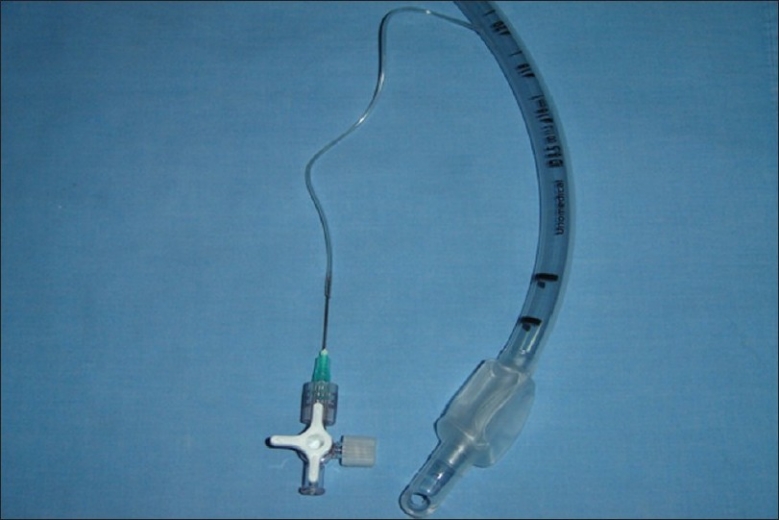
The needle is inserted into the severed inflating tube. Opening the stopcock to only the syringe and needle permits the cuff to be inflated

**Figure 5:**
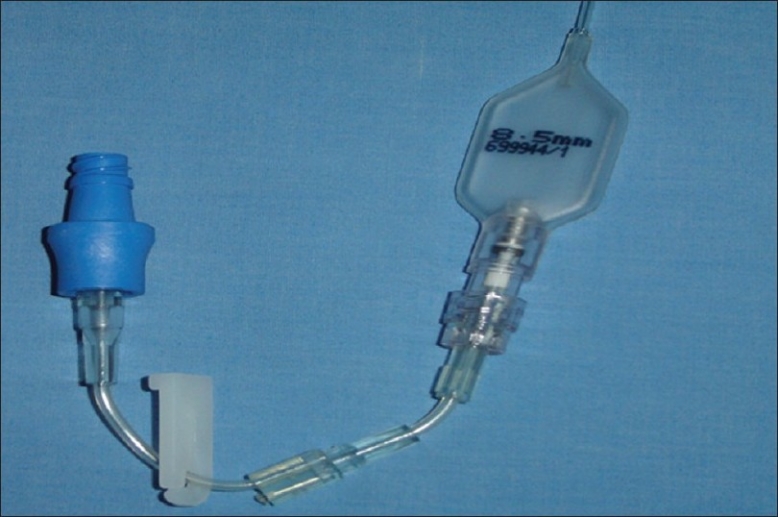
T connector can be connected to the pilot balloon to function as a secondary valve for the system

**Figure 6:**
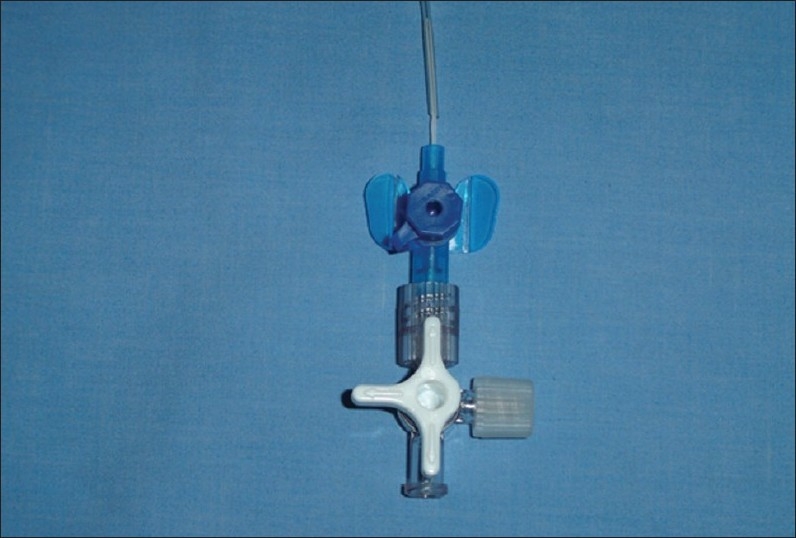
A 22 guage intravenous catheter is threaded directly into the lumen of inflation tube

We would like to give some suggestions regarding tube fixation and some temporary remedial measures in case of leaky cuff if reintubation is not feasible:

1. The above incident reprimands everybody that correct basic techniques should not be ignored as they can lead to disasters in case of difficult intubation.
2. While fixing the tube with adhesive plaster, it should not be overlapped multiple times, as it can lead to leaking cuff as in our case and also make its removal difficult during extubation [Figure 1].
3. The basic principle of traction and countertraction should be followed while securing the tube [Figure 2].
4. Even in case of simple intubations, junior doctors should be supervised and trained for securing the tube.
5. In case of difficult intubation and if the inflation tube is cut at the level of entry into tracheal tube, the technique described by Sprung Juraj[6] may be used. He used the inflation tube of an unused ETT, and joined to remnant section of the inflation tube on the patient's ETT using a 0.5-1-inch section of a standard hypodermic needle [Figure 3].[6] Fisher also described a similar technique wherein cut ends of the inflation tube were pulled together over the needle. The inflation tube was sprayed with tincture benzoin and wrapped with plaster.[7] In case of cracked one-way valve or cut pilot balloon or inflating tube, Jims Sill described a technique that can be used to reinflate the cuff.[1] The assembled device consists of a 10-ml syringe, a three-way stopcock, and a needle. The needle is inserted into the severed inflating tube. Opening the stopcock to only the syringe and needle permits the cuff to be inflated without the monitoring of pressure [Figure 4]. Watson also gave some simple solutions for leaking ETT cuffs due to incompetent pilot balloon valve.[8] T connector can be connected to the pilot balloon to function as a secondary valve for the system [Figure 5]. The plastic clip of the “T” connector can be loosened to add air to the system and then clamped to keep it pressurized. If the cuff still leaks, the problem may be a hole in the pilot balloon. If this is suspected, the pilot balloon is cut from ETT and a 22-guage intravenous catheter that has been moistened with alcohol is threaded directly into the lumen of inflation tube. Alcohol helps lubricate the catheter and then cements it in the tube after evaporating. The other end of the catheter may be attached to either stopcock [Figure 6] or T connector to keep the cuff pressurized.

## CONCLUSION

We have reported a case of leaking endotracheal cuff due to faulty fixation of tube. Securing the tube is a basic maneuver, yet if done improperly can lead to disasters. The basic principle of traction and countertraction should be followed for ETT fixation and multiple overlaps of adhesive plaster should be avoided. In case of difficult intubation or situations wherein reintubation is not possible, simple techniques described earlier can be used to reinflate the cuff.
